# An overview of recent applications of computational modelling in neonatology

**DOI:** 10.1098/rsta.2010.0052

**Published:** 2010-06-13

**Authors:** Luiz C. Wrobel, Maciej K. Ginalski, Andrzej J. Nowak, Derek B. Ingham, Anna M. Fic

**Affiliations:** 1 School of Engineering and Design, Brunel University, Uxbridge UB8 3PH, UK; 2 ANSYS UK Ltd, Sheffield Business Park, Europa Link, Sheffield S9 1XU, UK; 3 Institute of Thermal Technology, Silesian University of Technology, Konarskiego 22, 44-100 Gliwice, Poland; 4 CFD Centre, University of Leeds, Leeds LS2 9JT, UK

**Keywords:** computational fluid dynamics, heat and mass transfer, neonatology, incubators, radiant warmers, oxygen hoods

## Abstract

This paper reviews some of our recent applications of computational fluid dynamics (CFD) to model heat and mass transfer problems in neonatology and investigates the major heat and mass-transfer mechanisms taking place in medical devices, such as incubators, radiant warmers and oxygen hoods. It is shown that CFD simulations are very flexible tools that can take into account all modes of heat transfer in assisting neonatal care and improving the design of medical devices.

## 1. Introduction

The maintenance of an optimal thermal environment is regarded as a priority in neonatology. While full-term and healthy neonates are able to regulate their body temperature, premature and sick infants may often have difficulties keeping their body temperature at a constant level without external assistance. Owing to this immaturity of their thermoregulation system, they can suffer from cold stress and hypothermia, increasing the morbidity and mortality of premature and sick newborns (Lyon 2006; Knobel & Holditch-Davis 2007). For this reason, maintenance of the neonates’ bodies within a narrow temperature range is essential for their survival and growth. This temperature range is a thermal neutral (optimal) environment, which can be defined as ‘any set of environmental conditions that results in an optimal skin temperature which corresponds to a minimum resting metabolic rate’ (Malin & Baumgart 1987). Maintaining a thermal neutral environment in the case of very low-birth-weight infants is now routine practice in neonatal nurseries.

Medical devices to assist the thermoregulation of neonates include incubators, radiant warmers and heated mattresses, the first two being of common use in hospitals. Incubators were invented first, and provide an enclosed environment with warm air circulating inside the device. Radiant warmers are open devices, consisting of a radiant heater placed above a neonate lying on a crib. The main advantage of these devices over incubators is the ease of access to the neonate, which enables various medical interventions. The main drawback is that they increase evaporative heat losses, which may result in dehydration in the case of very premature babies.

Oxygen hoods are commonly used devices to deliver supplemental oxygen to neonates. An air–oxygen blender is often used with oxygen hoods to administer gas when a precise dose of oxygen is required. Hoods are a versatile method of oxygen delivery that can be used on neonates in an incubator or under a radiant warmer (Wyka *et al*. 2001). However, great care and constant monitoring must be in place, as delivering a cold, wet mist to the infant’s face will chill the infant, causing cold stress. Conversely, delivery of an overheated gas has the potential to induce hyperthermia, dehydration or pulmonary burns (Wyka *et al*. 2001).

Computational fluid dynamics (CFD) has been greatly developed over the last decade, mostly due to the rapid advance of computer technology. It is now possible to simulate complex scientific problems including several combined processes taking place simultaneously. CFD techniques have been successfully used to describe the thermal interaction between the human body and its surrounding environment (e.g. Murakami *et al*. 2000; Makinen *et al*. 2001; Sorensen & Voigt 2003). However, most of these studies were undertaken with reference to adults, whose physical shape, thermophysiological properties and thermoregulatory processes are different from those of infants (Strand 1980; Bach *et al*. 1996; Guyton & Hall 2000). Studies investigating physical processes occurring inside closed environments, such as incubators, have mostly been carried out through experimental techniques (Wheldon & Rutter 1982; Wasner 1994; Bolton *et al*. 1996; Rennie & Roberton 2002). CFD simulations can be very useful in many situations, as they easily allow parametric testing, can provide a wealth of data and can be of assistance in the design of more efficient, safe and reliable medical equipment.

This paper reviews some of our recent applications of CFD to problems in neonatology, and discusses the mechanisms of metabolic heat generation and sensible and latent heat losses, including heat conduction, convection, radiation and evaporation. The paper follows previous studies by the authors (Ginalski *et al*. 2007, 2008; Fic *et al*. 2008, 2009), in which CFD techniques were applied to study the air flow and heat transfer in medical devices such as incubators, radiant warmers and oxygen hoods. It also discusses recent studies in modelling brain cooling techniques for hypothermic interventions. Other possible applications of CFD simulations in neonatology may include studies on the optimization of respiratory support systems (Yamada *et al*. 2008).

## 2. Thermoregulation of neonates

Body heat is exchanged with the environment by different modes of heat transfer, including conduction, convection, radiation and evaporation. The metabolic heat generated inside the body is transferred by conduction through the tissue and convection through blood flow to cooler regions such as the skin surface and the respiratory tract. Radiation in the form of electromagnetic waves is significant in incubators. Evaporation consists of transepidermal water loss from the skin surface and water loss from the respiratory tract. The heat needed for water to evaporate is taken from the skin, resulting in a decrease in the body temperature. Heat loss is a particular problem for very small and premature newborns because of their high ratio of body surface area to body mass (Boxwell 2000).

Premature newborns have much lower energy resources than full-term infants, and this can lead to a negative energy balance if the heat losses are higher than the metabolic heat production. Furthermore, their bodies include a higher amount of water than full-term infants, and water is negatively related to energy content (Hulzebos & Sauer 2007). Premature newborns also have higher energy demands, and it is thus crucial that they are provided with the necessary energy and nutrient intake (Hulzebos & Sauer 2007). The additional heat is provided by medical devices such as incubators and radiant warmers.

Incubators are built with an enclosed volume to which externally heated air is supplied. The heating power can be controlled manually or by a thermostatic servo-control maintaining the skin, or the air temperature, at a desirable level (Bell 1983). A major concern when using incubators is the radiant heat loss of the neonate to the cold walls of the device. The main remedy to this situation is the use of double-walled incubators, where the walls are separated by a layer of air. The inner wall is warmed to a temperature close to that of the air inside the incubator, substantially reducing radiant heat losses (Bell 1983).

The situation is more complex in the case of smaller neonates. They are characterized by a large evaporative heat loss and can become hypothermic even inside incubators. In such cases, it is possible to increase the air humidity by supplying warmed humidified air to the incubator (Merenstein & Gardner 2006). Most modern incubators are equipped with a system to provide a supplemental humidification in order to increase the humidity by up to 95 per cent (Bell 1983).

The main disadvantage of incubators is the limited access they provide to the neonate. This is especially inconvenient when nursing sick newborns that are in need of some treatment or intervention, as opening an incubator during treatment may cause a decrease of 3–5^°^C in the air temperature. Radiant warmers are normally used in such cases, as they are open and provide easy access to the newborn. Thus, radiant warmers can be used in intensive nursery. However, there are a few concerns when using radiant warmers, as they increase convective and evaporative heat losses from the neonate’s body (Bell 1983). Evaporative heat losses, in particular, cannot be as easily limited by raising the humidity as in incubators. In the case of premature babies, this may result in severe dehydration. One remedy for this problem is the use of plastic shields or wrappings to prevent the water loss and dehydration (Boxwell 2000).

The governing equations of fluid flow and associated phenomena can be derived through the assumption of conservation of mass, momentum and energy, and have been solved in the present study by means of the commercial CFD solver ansys fluent (Fluent 2006). The Boussinesq approximation was adopted for the buoyancy term in the momentum conservation (Navier–Stokes) equation.

The rate of heat transfer by conduction is small for a neonate lying on a foam mattress, following Wheldon (1982), and thus can be considered as negligible. Typical flow Reynolds numbers for incubators indicated that a transition to turbulence type of flow occurs within their main chamber. For this reason, the airflow in the devices is considered to be turbulent and the shear-stress transport (SST) *k*–*ω* turbulence model has been used in all simulations, with a correction for flows characterized by a low Reynolds number. Radiation heat exchange is calculated in the CFD solver by using the discrete ordinate method. Air is considered to be a transparent medium for radiation modelling. Detailed information is provided in Ginalski *et al*. (2007).

The standard version of the solver possesses several limitations that reduce its applicability to calculate processes crucial for analysing the thermal balance of neonates. Some of the components of the infant heat balance, including heat generation and dissipation within the infant’s body, and heat loss due to evaporation from the skin and the respiratory tract, cannot be solved by the standard version of the CFD solver. Hence, a series of developments were necessary to allow the calculation of all the components of the infant heat balance. A supplementary module, entitled Infant Heat Balance Module (IHBM), was then developed and fully integrated into the main solver via user-defined functions (Ginalski *et al*. 2008). The IHBM is accessible through the ansys fluent main solver via a drop-down window with an additional menu section that allows the user to provide the necessary input data for modelling the above extra terms in the infant heat balance. Typical data involve the metabolic contribution to the bioheat equation based on the empirical formula introduced by Brück (1961), the heat transferred with blood per unit volume of the tissue, and information on respiration data and oxygen consumption.

Most of the mathematical analysis carried out in bioheat transfer to date is based on the Pennes equation (Rubinsky 2006). Capabilities for solution of the bioheat equation are also available in the solver. Metabolic contributions can be included as distributed energy sources. Other effects also included as heat sources in the bioheat equation are the heat transferred with blood, moisture evaporation from the infant’s skin and latent heat of evaporation from the infant’s skin. Empirical approximations for modelling these terms are discussed in Ginalski *et al*. (2007, 2008), and have been implemented in the IHBM module to supplement the CFD solver.

## 3. CFD simulations

In general, most CFD applications follow the same basic methodology. The main differences are related to problem complexity, available computer resources, available CFD expertise and whether a commercially available CFD package is used or a problem-specific algorithm is developed. The steps necessary to complete a CFD simulation can be classified into three main groups as follows:

— preprocessing, which involves activities necessary to build and set up the computational model;
— processing, involving the solution of the system of differential equations describing the problem under consideration;
— postprocessing, which involves the presentation of results in numerical or graphical form.

In this paper, the validation of the CFD simulations is carried out by comparison with experimental measurements from the medical literature, whenever possible. The parametric capabilities of the proposed numerical techniques allowed the models to be adjusted to match some of the conditions during which the experiments and/or measurements took place.

The main objective is to develop benchmark models that could be properly validated against experimental data, and then used in further parametric studies to improve the design of medical devices or to explore new medical hypotheses, particularly where these are difficult to measure, thus reducing the need for clinical trials and/or animal testing. An example is the study of Ginalski *et al*. (2007) on the effects of adding an overhead screen to an existing incubator to decrease heat losses from the infant. The CFD calculations of Ginalski *et al*. (2007) were performed for a range of different air inlet temperatures and showed a decrease of radiative and convective heat losses when the overhead screen is present. CFD models can also be used in patient-specific studies to assist in the decision of the best course of treatment in each case.

### (a) Model geometry and discretization

For complex CFD simulations involving multiple geometric parts created in different applications, the geometry and grid-generation processes can be extremely challenging. Geometric representations of the neonates were built by using models in stereolithographic STL format modified in 3D Studio MAX (Murdock 2001) to represent the thermal manikins used in the experiments. Utilization of 3D Studio MAX combined with the wrapping technology available in the ANSYS TGrid (TGrid 5.0 2008) preprocessor proved to be a very efficient tool for generating complex and accurate models for the CFD simulations. It allowed the preprocessor to be used in a fully automatic way, thus substantially reducing the time required for completing this task.

**Figure 1.**
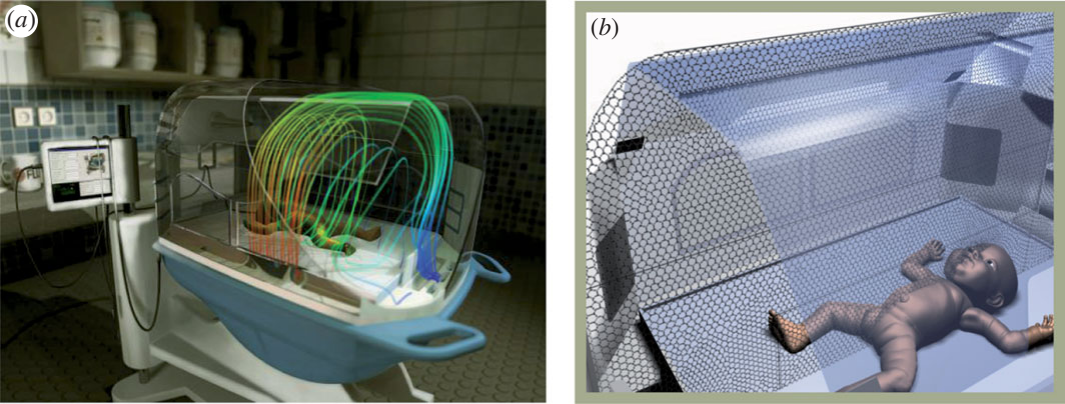
(*a*) Three-dimensional model of the Caleo incubator, with CFD results displayed as flow pathlines coloured by temperature; (*b*) polyhedral mesh for the Caleo incubator.

Several calculations with different mesh sizes have been performed to verify the accuracy of the simulations, as described in Ginalski *et al*. (2007). Quantities like the heat transfer due to radiation and the total heat transfer exchanged between the infant and the surrounding environment were monitored and compared. The meshes analysed were composed of tetrahedral elements, with typical converged meshes consisting of 1 500 000 elements. A typical model is shown in figure 1*a*, where the CFD results for the simulation of a double-walled incubator are shown as flow pathlines coloured by temperature. More recent simulations employed polyhedral meshes available in the ansys fluent solver (Ginalski *et al*. 2008), which provide better convergence and stability during the calculations of complex physical processes. A typical polyhedral mesh for an incubator is shown in figure 1*b*.

### (b) Dry heat losses in incubators

Simulations of dry heat losses from a neonate within an incubator were presented in Ginalski *et al*. (2007, 2008). Verification of the CFD simulations was performed by comparison with the results of Elabbassi *et al*. (2004), who described experiments to assess the heat losses for small neonates using two anthropomorphic thermal manikins, representing newborns of 900 g and 1800 g. During the experiment, dry heat losses were measured from the six body segments of the manikins. The effect of different environmental thermal conditions was investigated with air temperatures ranging between 29^°^C and 35^°^C. However, since only dry heat losses from the infant’s body were investigated in Elabbassi *et al*. (2004), the evaporation and respiration processes were not considered in the numerical simulations.

The results of these simulations were compared with those measured by Elabbassi *et al*. (2004), who provided equations, obtained from linear regression, to estimate the dry heat loss (by conduction, convection and radiation) for each of the manikins. The same manikins of 900 g and 1800 g were considered in the CFD simulations, for temperature values of 32^°^C, 34^°^C and 36^°^C, nursed in the Caleo Neonatal Incubator (2002), which is a more advanced neonatal unit than the BioMS C 2750 Incubator (BioMS, Toulouse, France) used in the experiments, for which no detailed data were available. The results of the simulations displayed similar trends to those observed in the experiments in all cases. However, the comparison is qualitative rather than quantitative since different incubators were used in the two analyses.

### (c) Latent heat losses in incubators

 Wheldon & Hull (1985) described a series of experiments investigating the metabolic heat generated by an infant’s body and the evaporative heat losses from the infant’s skin. Their results clearly demonstrated that, for premature infants with less than 30 weeks gestation, heat losses due to evaporation exceed 30 per cent of the total metabolic heat generated in all analysed cases. Additionally, according to Agren *et al*. (1998), the evaporation losses from the infant’s skin in certain situations can be as high as 60 g (m^2^ h)^−1^. Such a high evaporation rate would result in 3.6 W of latent heat loss caused by the evaporation process for the 900 g infant analysed in the previous validation case. At the same time, the metabolic heat generated by the infant, based on the equation proposed by Brück (1961), would only result in 1.5 W. This particular example indicates the importance of evaporation heat losses in the total infant heat balance. In the case of term infants born after 32 gestation weeks, heat loss due to evaporation is less intensive. However, in lower-humidity environments, it can still significantly influence the overall infant heat balance (Hammarlund *et al*. 1977; Sedin *et al*. 1983).

Water loss from the infant’s skin is only part of the total water losses due to evaporation. A similar process occurs within the lungs during respiration. The IHBM module possesses the capability of calculating both the respiratory and transepidermal water losses from the infant’s body.

## 4. Results and discussion

A series of validation tests has been performed based on extensive studies of Hammarlund *et al*. (1977) and Sedin *et al*. (1983), who provided detailed information on the weight, length, mass and gestation age for a group of 34 infants analysed during their experimental studies. From this group, 19 infants were selected for the simulations, varying from 25 to 33 weeks gestation. More information on the test cases analysed and discussion of the results is given by Ginalski *et al*. (2008).

The validation of results has been performed with respect to experimental measurements available in Hammarlund *et al*. (1977) and Sedin *et al*. (1983). Unfortunately, those articles did not include all the data required to reconstruct accurate representations of the infants analysed, and the properties of the incubator and the environment in which they were nursed. Hence, the comparison of the CFD results with data published in these articles can only provide limited validation of the CFD model.

The experiments of Hammarlund *et al*. (1977) examined the evaporation rate from an infant’s skin at different ambient air conditions. The latent heat loss was calculated as the product of evaporation rate and latent heat of evaporation of water. The infant selected for the CFD simulations had the following characteristics: weight 1900 kg, length 0.44 m, gestation age 33 weeks (Hammarlund *et al*. 1977).

The mean skin temperature for the infant was calculated in the CFD simulations as a weighted average of each computational face representing the infant’s skin. The body core temperature was calculated as the maximum temperature within the infant’s body. The CFD results for the mean skin temperature and the body core temperature were 35.4^°^C and 36.8^°^C, respectively, while the corresponding data obtained by Hammarlund *et al*. (1977) were 35.3±0.1^°^C and 36.3±0.1^°^C.

CFD simulations were then performed for nine different ambient conditions, in which the air humidity ranged from 20 to 60 per cent. Air temperature and velocity are kept constant in all simulations. The results from the CFD simulations are compared with the experimental data in table 1. It must be reiterated that different incubators were used for the simulation and the experiments, and also that there are several missing parameters in Hammarlund *et al*. (1977) related to the velocity field within the incubator and the external environment. Taking this into account, it can be concluded that the algorithm used by the IHBM module to calculate latent heat losses due to moisture evaporation from the infant’s skin provides accurate results. Once again, the Caleo Neonatal Incubator (2002) was used in the CFD simulations instead of the AGA MK41 Incubator (AGA Medical, Lidingo, Sweden) used in the experiments, for which detailed data were not available, justifying the reduced heat losses. Although the accuracy of the CFD simulation has been verified by mesh-independence tests, standard modelling and experimental errors may also play a part in explaining the differences between the results. However, the two sets of results are within the expected physiological ranges for the infants considered.

**Table 1.** Comparison of evaporation heat loss results.

	evaporation heat loss (W)	
relative air humidity (%)	Hammarlund *et al.* (1977)	CFD calculations
20	0.84	0.70
25	0.79	0.66
30	0.75	0.62
35	0.70	0.58
40	0.65	0.55
45	0.61	0.50
50	0.56	0.46
55	0.51	0.43
60	0.47	0.39

The next validation test case concerns respiration heat losses. To validate this last remaining component of the infant heat balance, four different cases were investigated by making use of the full capabilities of the IHBM module. The validation tests were performed on the basis of experimental data for respiratory heat losses obtained by Sedin *et al*. (1983), who analysed the influence of humidity and temperature on the insensible water loss from the infants using the direct calorimetric method. Information including infant weights, size and nursing position has been used as parameters to automatically generate representative infant models and is presented in table 2, together with other parameters necessary for modelling the respiration process.

**Table 2.** Physical characteristic of the infants.

infant number	weight (kg)	length (m)	tidal lungs volume (ml)	lungs dead space (ml)	respiration rate (bth min^−1^)	respiration flow rate (ml min^−1^)
1	3.242	0.50	22.7	8.1	52	379.6
2	3.207	0.49	22.4	8.0	48	345.6
3	3.277	0.52	22.9	8.2	50	367.5
4	3.196	0.51	22.4	8.0	57	410.1

In the first stage, deep body and skin temperatures have been compared with the corresponding experimental data (Sedin *et al*. 1983), as presented in table 3. The maximum deep body temperature difference between the CFD and experimental results was less than 0.6^°^C, while the maximum difference for the average skin temperature was less than 0.8^°^C. These results indicate good accuracy of the CFD calculations, in line with the previous cases. The difference between the experimental and numerical results can be justified by the lack of some of the data necessary to fully describe the model in Sedin *et al*. (1983).

**Table 3.** Comparison of body temperature results.

	Sedin *et al.* (1983)		CFD calculations	
infant number	mean skin temperature (^°^C)	body core temperature (^°^C)	mean skin temperature (^°^C)	body core temperature (^°^C)
1	35.4	36.7	35.3	37.3
2	36.0	36.9	36.7	37.4
3	35.7	36.7	34.9	37.0
4	35.5	36.6	35.9	36.9

The average contribution of the respiration heat losses within the total heat losses caused by moisture evaporation from the respiratory tract and from the infant’s skin is 42 per cent for the experimental results and 45 per cent for the CFD results. These numbers indicate the importance of properly modelling respiration heat losses.

### (a) Air flow and heat transfer in a radiant warmer bed

**Figure 2.**
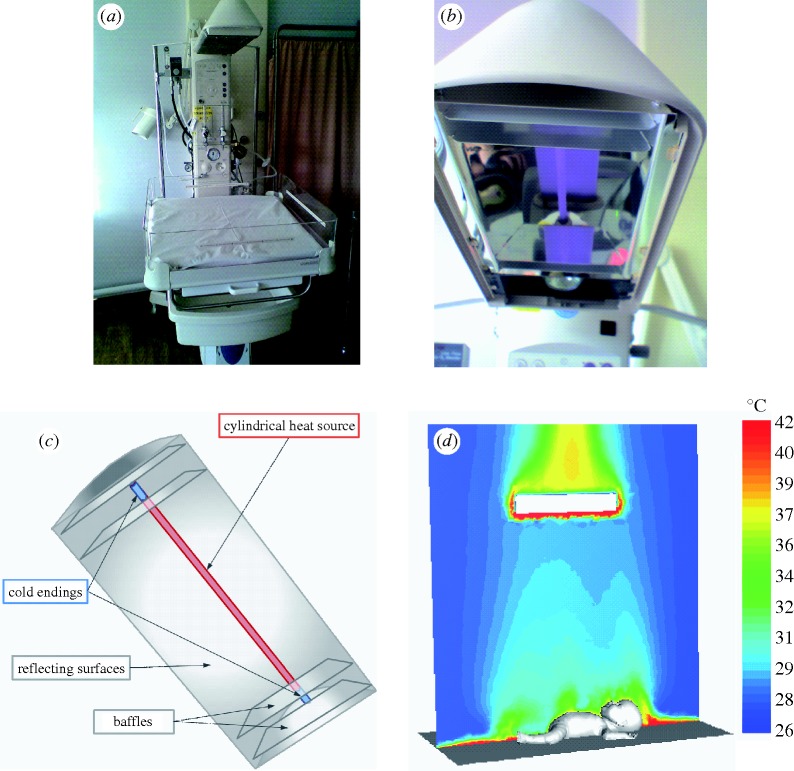
(*a*) General view of the Fisher & Paykel CosyCot radiant warmer at Hammersmith Hospital; (*b*) bottom view of the lamp; (*c*) geometry of the lamp incorporated into the model; (*d*) temperature field obtained from the CFD model with the neonate in the spread position.

The initial data for this series of simulations were based on the information found in LeBlanc & Edwards (1985) for a KDC 660 radiant warmer. More recently, collaboration has been initiated with Hammersmith Hospital in London, which is equipped with CosyCot infant warmers (Fisher & Paykel Healthcare Ltd., Maidenhead, UK). A photograph of this radiant warmer taken at Hammersmith Hospital is shown in figure 2. As can be observed in figure 2*a*, the radiant warmer contains the neonate’s crib and a radiant lamp placed above it. The lamp is better visible in figure 2*b*, while the geometry of the lamp built for the purpose of numerical simulations is shown in figure 2*c*. The radiant lamp consists of a cylindrical heat source surrounded by parabolic reflecting surfaces. The endings of the lamp are ‘cold endings’, as no heat generation occurs there. Baffles are placed perpendicular to the tube and the parabolic reflector to shield the rear of the lamp from the radiant heat. The importance of the presence of these baffles will be investigated in future research.

Temperature measurements on a CosyCot infant warmer, both with and without a neonate, were taken at Hammersmith Hospital using a Flir thermal camera. Fisher & Paykel has also provided some data on the emissivity of the radiant warmer surfaces, as well as the temperatures of the radiating element inside the lamp. In addition, they have provided the dimensions of the radiant lamp, making it possible to create a realistic model as shown in figure 2*c*.

Contrary to incubators, radiant warmers are open devices with no external walls. Because the modelling of the whole room with the radiant warmer inside would be too computationally expensive, the domain for the numerical simulation had to be limited by artificial boundaries, with boundary conditions of pressure inlet and pressure outlet. Several tests were performed to find the optimum dimensions of the computational domain for which the artificial boundaries would not significantly interfere in the air flow and heat transfer between the radiant warmer and the newborn. A domain with dimensions of 2×2×2.17 m (length×width×height) was found to provide a good compromise between numerical accuracy and computer efficiency. Moreover, according to the dimensions of the CosyCot warmer, the mattress is square with side length of 61 cm, placed at a height of 90 cm. The lamp is situated 71 cm above the top surface of the mattress. The modelling of the lamp is crucial in this model. Namely, the parabolic reflector surrounding the heat source tube reflects the radiation in a mirror-like manner. Together with locating the tube in the focal position of the parabola, the mirror-like reflection of the radiator enables the heat radiation to be directed towards the infant rather than being scattered in all directions.

The calculations have to be performed in a particular manner to improve the convergence of the CFD solver during the iterative process. Initially, only natural convection from the baby is considered, with the radiator off and no radiation modelled. In the next step, radiation is included in the model, while in the final step the radiant warmer is switched on. Transient calculations are performed to improve convergence, leading to a steady-state solution.

The temperature field presented in figure 2*d* shows the convective plume created over the neonate (in the symmetry plane of the domain) due to natural convection. The temperature scale in this figure has been lowered to 42^°^C in order to visibly present the convective plume. However, the maximum temperatures occurring at the bottom of the radiant lamp are much higher, where the air is in contact with the surface of the lamp. The natural convection phenomenon is difficult to model because of the instability of this physical setup. For this reason, at this stage of research, many simplifications have been made to the model. The research on radiant warmers is still on-going and only preliminary results have been obtained so far. More details of some of the different models created at this stage can be found in Fic *et al*. (2009).

The results presented in Fic *et al*. (2009) are in qualitative agreement with the measured data. However, a more detailed model of the radiator lamp is currently being tested, which includes the correct geometry of the radiating element for the Fisher & Paykel CosyCot radiant warmer surrounded by the parabolic reflecting surfaces. The values of the internal emissivity and the diffuse fraction for the lamp and the reflector were given by Fisher & Paykel. It is expected that these and other changes to the radiation modelling will provide numerical results in closer quantitative agreement with the measured data.

### (b) Modelling of the respiration process in an oxygen hood

The ability to model respiration as a transient process is very important in some situations, e.g. those including oxygen concentration studies and drug delivery. For this reason, a series of simulations has been performed in order to investigate the distribution of oxygen concentration under an oxygen hood. The relatively small dimensions of this device enhanced the importance of the respiration process on the overall air flow pattern surrounding the infant. The computational domain for the calculations included only the head of the infant placed under the oxygen hood. The hood geometry was created based on information from the manufacturer. However, it must be stressed that the numerical simulations have not been validated and the manufacturer only provided a brief description of the model.

**Figure 3.**
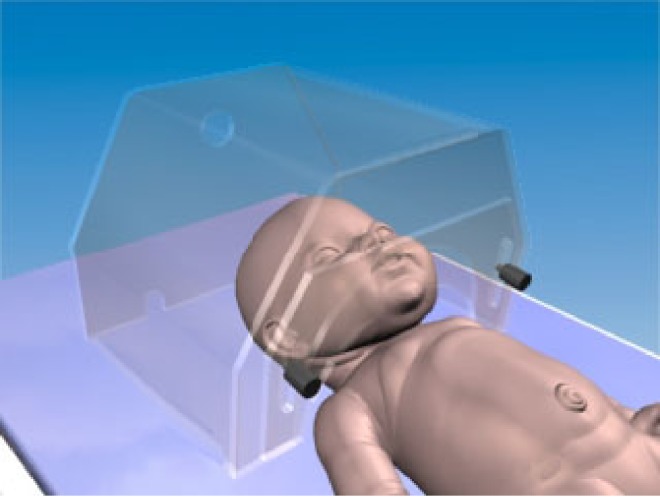
Geometrical model of a neonate nursed under an oxygen hood (reproduced from Ginalski *et al*. (2008) with permission from IOP Publishing).

The quality of the mesh was, once again, verified by mesh-independence tests, which focused on node distribution, cell shape and smoothness. As usual, the mesh was more refined near the salient features of the flow (such as boundary layers, separated regions, shear layers). Rapid changes in the cell volume between adjacent cells were avoided to minimize truncation errors. The aspect ratio of the cells was kept at less than 5 : 1, and their skewness was also controlled to avoid the appearance of degenerated cells. The final computational domain is presented in figure 3, and the final mesh had a total of 1 320 000 polyhedral elements.

The *k*–*ε* turbulence model was used in the calculations due to the high Reynolds numbers at the exhaled stage of breathing. All calculations were performed for the same infant characteristics as in the previous validation case regarding respiration heat losses. In order to limit the number of cells in the simulation, the centre plane was considered as a plane of symmetry. Air was considered to be a multispecies mixture of oxygen, nitrogen, carbon dioxide and water vapour. An incompressible ideal gas law was employed to take into account density variations caused by temperature differences.

Velocity boundary conditions were set at the inlets to the computational domain and at the infant nostrils, using the information provided by Rennie & Roberton (2002) and by Ginalski *et al*. (2008). The respiration pattern was defined as a function of the respiration rate, the lungs’ tidal volume, the lungs’ dead space, the density of the exhaled air and the nostrils’ surface area, according to an equation given by Guyton & Hall (2000).

**Figure 4.**
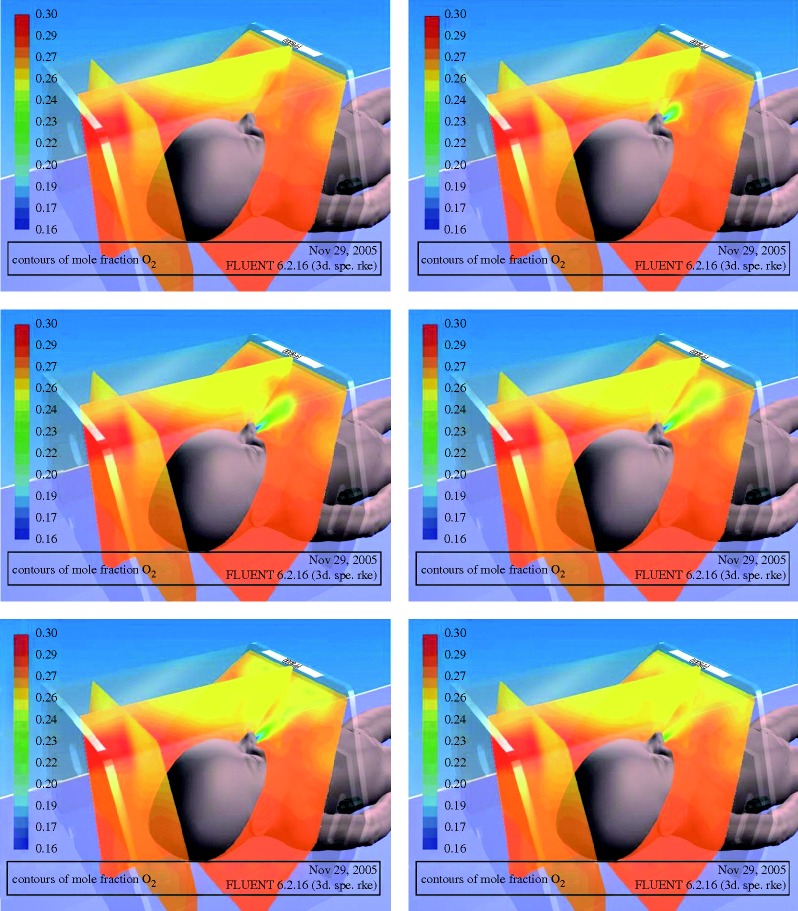
Contours of oxygen mole fraction plotted at different stages of the respiration cycle (adapted from Ginalski *et al*. (2008) with permission from IOP Publishing).

The numerical simulation was performed for a time interval of 25 min of regular breathing, and aimed at demonstrating the potential capabilities of CFD techniques for transient modelling of the respiration process. Hence, the results obtained at this point have not been validated. However, several important conclusions can be obtained. For example, the oxygen concentrations in the air provided to the oxygen hood and in the air inhaled by the infant are considerably different. The respiration pattern and other related parameters will also influence the oxygen concentration in the inhaled air. CFD techniques can help in determining the level of oxygen concentration and the optimum position of the infant’s head under the hood. Figure 4 shows a sequence of snapshots of the oxygen mole fraction during the expiration stage. The snapshots have been taken at the 25th minute of the simulation. Similar distributions were also obtained for carbon dioxide. In comparison with oxygen, carbon dioxide dissipates quickly within the domain. Hence, the concentration profile of this gas is much more homogeneous, confirming that the oxygen hood is properly ventilated.

## 5. Hypothermic treatment of perinatal asphyxial encephalopathy

Our current research aims at developing an advanced thermoregulation model for neonates, which will include the modelling of the temperature distributions inside the neonate’s head. The model will allow numerical simulations of brain cooling as a contribution to the investigation on the use of hypothermia for the treatment of perinatal asphyxial encephalopathy.

Perinatal asphyxia causing moderate or severe encephalopathy occurs in approximately two of 1000 births. Causes include damage to the umbilical cord, detachment of the placenta or rupture of the uterus during labour. There is an increasing risk of death or neurodevelopmental abnormalities with more severe encephalopathy. In full-term newborns, perinatal asphyxia may account for up to 30 per cent of cases of cerebral palsy. At present there is no specific treatment for asphyxia other than stabilization with treatment to reduce seizures. Pilot studies of head cooling combined with mild whole-body hypothermia and of moderate whole-body cooling to 33–34^°^C have been reported in infants with encephalopathy, with the aim of determining whether the use of body cooling following perinatal asphyxia is a safe treatment that will improve survival and reduce neurological and neurodevelopmental impairments (Edwards & Azzopardi 2006).

Previous theoretical research suggests that it is uncertain whether head cooling alone is effective in lowering deep brain temperature. According to Van Leeuwen *et al*. (2000), ‘If the head is cooled while maintaining normal systemic temperature, the temperatures in deep brain structures remain far above the target temperature for mild hypothermia’. This statement is further supported by the results reported by Dennis *et al*. (2003), where numerical simulations of brain cooling were carried out by considering ice packs applied to head and neck, as well as using a head-cooling helmet. It is reported by Dennis *et al*. (2003) that ‘It was found that neither of these cooling approaches satisfies the 33^°^C temperature within 30 mins’ criterion.

Results of studies by Zhu & Diao (2001) and by Diao *et al*. (2003) suggest that it should theoretically be possible to cool the brain tissue much faster when the brain is highly perfused. They also suggest that another factor that may contribute to the slow cooling of the brain in previous trials is the thermal resistance between the coolant and the scalp.

An alternative technique involves whole-body cooling. This technique would allow the use of much simpler and cheaper devices such as cooling mattress to achieve the same brain cooling results.

 Edwards & Azzopardi (2006) discuss extensive experimental data resulting from clinical trials for the CoolCap project (Gluckman *et al*. 2005), from the National Institute of Child Health and Human Development (NICHD) (Shankaran *et al*. 2005) and from Eicher *et al*. (2005), and conclude that: ‘Well constructed and carefully analysed trials of hypothermic neural rescue therapy for infants with neonatal encephalopathy have recently been reported. The data suggest that either selective head cooling or total body cooling reduces the combined chance of death or disability after birth asphyxia. However, as there are still unanswered questions about these treatments, many may still feel that further data are needed before healthcare policy can be changed to make cooling the standard of care for all babies with suspected birth asphyxia.’

Substantial theoretical extensions to our current model are required for brain cooling studies, with the development and implementation of suitable techniques to predict detailed temperature distributions in the deep brain via the combination of a simplified whole-body thermoregulatory technique with an advanced head cooling model.

The regulatory processes that control the cerebral blood flow depend on the mean arterial blood pressure and on physiological parameters, including the partial pressure of oxygen, the partial pressure of carbon dioxide and the cerebral metabolic rate of oxygen consumption, which is also affected by the tissue temperature. The effect of these regulatory mechanisms on cerebral blood flow has been confirmed by the experimental findings of Payne *et al*. (1963).

The new thermoregulation model for neonates will incorporate the effect of cerebral blood flow on metabolic heat rate via the Pennes bioheat equation. The consideration of temperature-dependent metabolic heat generation is also important in this case since it is known that the assumption of constant metabolic heat generation is only valid for healthy conditions (Xu *et al*. 1999). A simple analytical solution of the Pennes equation with temperature-dependent metabolic heat generation in which the brain was modelled as a hemisphere of cerebral tissue with overlaying layers of skull and scalp, and blood perfusion was assumed to reduce to 20 per cent of its normal value during ischaemia, provided some insight into cooling penetration and the characteristic cooling time for head helmets and ice packs (Zhu & Diao 2001; Diao *et al*. 2003). The dependence of both the blood perfusion and metabolic terms of the bioheat equation on temperature, tissue type and physiological parameters will be considered in the model via an autoregulation mechanism based on an intracranial dynamics model developed by Thoman *et al.* (1998).

The above developments have been initiated, and it is predicted that the resulting thermoregulation model for neonates will then be coupled with the CFD model for heat exchange with the ambient environment. The complete model will be validated with clinical data from the TOBY trial, which enrolled 325 infants (Azzopardi *et al*. 2009), resulting in an advanced, patient-specific computer model capable of supporting neonatologists and paediatricians on a number of important clinical applications.

## 6. Conclusions

This paper presented an overview of CFD studies of the heat balance in neonates. The CFD simulations have been validated against experimental measurements from the medical literature, and proved to be reasonably accurate in estimating the core and skin temperatures of infants nursed within incubators. The study also demonstrated the CFD capabilities of simulating the transient processes occurring within oxygen hoods. These studies aim at optimizing the design of medical devices used in neonatology, in order to improve their performance.

A recently started research investigates design improvements to radiant warmers, which are used to nurse unstable neonates requiring continuous interventions. As the infants are nursed in an open environment, a large amount of radiant heat is lost to the surroundings. Furthermore, as the neonates may be observed under a radiant warmer for several hours, water losses may be up to 50 per cent higher than in incubators, increasing the risk of dehydration (Merenstein & Gardner 2006). The usual remedy to decrease water loss is to wrap the infant in a plastic shield, but this impairs the access to the neonates. It is expected that CFD simulations may provide a better understanding of the problem, leading to better procedures for the management of radiant warmers.

A further new study aims at helping to determine whether the use of body cooling following perinatal asphyxia is a safe treatment that will improve survival and reduce neurological and neurodevelopmental impairments. These computer trials will test the feasibility of brain cooling using different types of cooling mattresses and body wrappers, and have the potential to reduce the number of clinical trials normally required for this type of study.
